# Supporting Young Children’s Self-Regulation Through Nature-Based Practices in Preschool

**DOI:** 10.3390/bs14111013

**Published:** 2024-10-31

**Authors:** Julie Ernst, Hannah Stelley

**Affiliations:** 1 Applied Human Sciences, College of Education and Human Service Professions, University of Minnesota Duluth, Duluth, MN 55812, USA; 2 Prairie Woods Environmental Learning Center and Nature Preschool, Spicer, MN 56288, USA; hannah.s@prairiewoodselc.org

**Keywords:** nature preschool, outdoor play, nature play, self-regulation, executive control, attention, impulse control

## Abstract

Self-regulation is a crucial skill in early childhood, due to its influence on school readiness and success, as well as its foundational role in promoting wellbeing across the lifespan. Additionally, self-regulation is malleable, particularly during early childhood. This study investigated the impact of nature-based practices on preschoolers’ self-regulation. The Preschool Self-Regulation Assessment (PSRA) and PSRA Assessor Report were administered to 115 children from nine preschool programs at the beginning and end of the school year. While further research is needed, results suggest the potential for nature-based practices to support SR, in particular hot executive function, and particularly in children from lower socio-economic backgrounds in public preschool. This study contributes to the literature regarding effective practices for supporting self-regulation development in young children and adds to the growing body of research surrounding the impact of nature play on child development and school readiness.

## 1. Introduction

### 1.1. Self-Regulation and Its Significance in Early Childhood

Self-regulation (SR) is among the aspects of early development that are strongly predictive of later outcomes across the lifespan. SR is the ongoing and dynamic modulation of emotion, cognition, and behavior, directed toward adjusting mental and physiological states adaptively to a given context [1]. It is composed of top-down (deliberate, volitional) processes of cognitive control and higher-level executive functions and bottom-up (automatic) processes, which together alter emotion, behavior, or cognition to enhance adaptation or achieve an explicit or implicit goal or goal state [1]. In other words, SR is what allows one to control one’s thoughts, emotional reactions, behaviors, and social interactions, even when impulses run contrary to the goal at hand or a future goal [2].

SR is of great interest across many fields, including education, psychology, developmental science, psychiatry, sociology, neuroscience, and medicine, due to its predictive links to academic achievement, educational attainment, health, and well-being [3]. However, it is a broad and challenging construct to define. There is much disagreement regarding its definition and structure across disciplinary lines of inquiry, which is made even more challenging due to the complex and dynamic nature of the process and systems involved. Some fields have focused on the cognitive top-down aspects of SR. For example, within social and personality psychology, effortful control (cognitive control in the service of SR) has been emphasized [4], and neuroscience and cognitive science have focused on the executive functions that control attentional, cognitive, and behavioral processes in goal-directed behavior [5]. There are also bottom-up, non-cognitive aspects of SR that are elicited by sensory stimuli and are linked with ‘feed-forward’ neural signaling [1]. Additionally, SR has distinct cognitively mediated (cool) and affectively or motivationally mediated (hot) processes [6]. (See [1] for a review of terminology and linkages among SR-related constructs, as well as a descriptive framework for linking disciplinary studies).

Despite disagreement regarding its conceptual and theoretical structure, SR is generally accepted as a multifaceted construct, with wide agreement regarding the importance of SR in early childhood due to its malleability and predictability of many short- and long-term outcomes. Children’s SR upon school entry is integral to their school functioning and adaptive classroom behavior. SR allows children to maintain attention, follow social rules, regulate emotions, suppress impulses, and focus on goal-directed stimuli [7]. Research also suggests SR has long-lasting impacts on children’s academic success. For example, children entering school with low SR were behind their high SR peers in reading and mathematics, with the gap widening between kindergarten and second grade and persisting through the elementary years [8]. Beyond its links to school readiness and academic performance [9], SR is closely related to school engagement and meta-cognition [10], aiding in skills like attentional control, problem-solving, persistence in challenging activities, and coping strategies for managing stress [11]. Further, supporting SR development in early childhood is an investment in later success, as not only is it a robust predictor of academic success and educational attainment [12] but it also has profound impacts on mental and physical health [13] and social competence in adulthood [14].

### 1.2. Supporting the Development of Self-Regulation

As described in [1], SR develops hierarchically across the lifespan, with lower-level capacities assembling into more complex ones, alongside the development of other physical and neural systems. Over time, these hierarchically organized capacities become reciprocally integrated, resulting in a regulatory system that draws upon the coordination of many processes across levels of functions [15]. Beginning around age three, children generally transition from a more reactive, co-regulated form of SR to more advanced, internal forms of SR that build upon children’s developing executive function and language skills [1], with children increasingly being able to draw upon, integrate, and orchestrate these multiple processes [8]. This development in SR, language, and executive function parallels prefrontal cortex development, which collectively supports more sophisticated forms of SR as children grow [16].

Early childhood is an important time for supporting the development of SR, as this is a period of rapid growth and architectural change in areas of the brain associated with SR. In addition to brain development, there are other varied and interacting influences on children’s SR development. A child’s regulatory abilities are also influenced by genetics, temperament, and environmental context [17]. Caregivers can be particularly influential on children’s SR development, modeling SR through warm, responsive relationships and buffering children from harmful or stressful experiences [18]. SR is also malleable, and interventions targeting SR skills can also effectively support SR development, particularly when accompanied by intrinsic motivation or extrinsic motivational support to enact SR skills [18]. Research suggests these interventions can lead to behavioral improvement, as well as corresponding changes in neural functioning [19].

Efforts to support SR development generally focus on interventions in early childhood education settings, given the prevalence of early childhood education and care alongside robust evidence of the potential for positive impacts [20]. Not only is early childhood a period of high malleability but it also coincides with a period when children experience sharp increases in the demands placed on their regulatory processes, particularly as they transition to school [19]. Additionally, with the significant relationship between SR and income [21], as well as childhood socioeconomic status (SES) more broadly [22], supporting SR development through early childhood education is particularly beneficial for children from lower SES families [23]. SR is an important protective factor, supporting children’s emotional and behavioral resilience to socio-economic disadvantage [24]. As such, it is crucial to find accessible and effective ways to support SR during early childhood [25].

In addition to the efficacy of targeted interventions provided to specific children for a particular reason, the literature suggests universal interventions provided to all children in an early childhood or school setting can be very useful for promoting SR development and preventing problems related to low SR. In a review of nearly 300 SR intervention studies, many different skill intervention approaches yielded positive outcomes of small to moderate effect sizes across different SR domains [26]. However, it is important to note that that these specific training effects may not generalize to other cognitive skills, and that without ongoing practice, benefits tend to diminish over time [27].

### 1.3. The Potential for Play in Natural Environments to Support Self-Regulation

Beyond skill-based interventions, play is widely acknowledged as an avenue for improving SR during early childhood [28]. Several reviews of play studies implicate the pretend and imaginative aspects of play as contributing to SR and/or related processes (e.g., [29]), while other studies have honed in on the social nature of play (e.g., [30]). Sociodramatic play may be particularly effective, with research suggesting social pretend play is predictive of gains in inhibitory control more than solitary pretend and social non-pretend play [31]. Additionally, the narrative aspects of sociodramatic play may be particularly influential on SR-related processes [32]. Physically active play has been correlated with SR and executive function, with play-oriented physical activity producing higher levels of SR than physical activity in non-play contexts [33]. Child autonomy and lack of adult structure also strongly contribute to SR [34]. Further, complexity and level of play also appear related to SR, with more complex pretend play [28] and mature (fully developed, advanced) play [35] associated with higher levels of SR. Unstructured quiet free play in the preschool years also is predictive of SR in later elementary years [36].

These characteristics of play align with nature play or child-led play in natural environments that is unstructured, freely chosen, and involves interactions with natural elements. Not only is there theoretical potential for nature play to promote SR development, there is also supporting empirical evidence of nature play’s influence on SR and related constructs. For example, in a study of 14 public preschools, nature play was effective in supporting preschoolers’ executive function skills (top-down SR processes) [37]. Preschools that incorporated some nature-based practices were significantly more effective than preschools that had little incorporation of nature-based practices, and this approach was particularly effective for supporting executive function in lower SES children [37]. Similarly, research has also suggested that play in natural playscapes supports preschoolers’ executive function skills, potentially due to the opportunities for problem-solving, self-planning and organizing, and risk-taking afforded by play in naturalized playscapes or green schoolyards [38]. Additionally, a recent study found that kindergartners’ use of green schoolyards supported SR development, with more frequent visits (daily v. weekly) and visits of greater duration associated with greater gains in SR [39]. Additionally, there is evidence suggesting that even short-term exposure to natural environments can be restorative for cognitive functioning [40] and enhance attentional functioning [41], which are underlying resources in the exhibition of SR. However, additional research is needed to understand the potential of nature-based practices to support SR development in young children, given the sensitive early childhood period, particularly for children from lower SES families. Further research would also aid in the current early childhood education landscape, with its emphasis on school readiness and concerns regarding nature play detracting from children’s readiness for and success in school [42].

### 1.4. Study Aim

The study at hand explored the influence of nature-based practices on preschoolers’ SR by comparing three preschool approaches that differed in the degree to which nature-based practices were incorporated (Less Nature vs. Blended vs. Nature). The aim of this study was to add to the research literature on effective practices for supporting SR, particularly in preschoolers from lower SES backgrounds. Additionally, the study aimed to add to the literature regarding the impact of nature play on child development and learning. The study was guided by the following research questions:

1. Which preschool approach (Less Nature vs. Blended vs. Nature) is most effective for supporting children’s SR, when accounting for age, gender, and SR pretest level?
2. Does the effectiveness of the preschool approach (Less Nature vs. Blended vs. Nature) for supporting SR vary by preschool setting (public vs. private preschools), and if so, which approach is most effective in public preschools primarily serving children of lower socio-economic backgrounds?

## 2. Materials and Methods

### 2.1. Participants

There were 115 preschool-aged children (three- to five-year-olds) from nine preschool programs who participated in the study. Participants’ gender and SES are reported in Table 1. Participating preschool programs included private (three preschool programs) and public schools (six preschool programs), all within the same local geographic area. The participating public preschool programs were from the same school district; in this district, there is an integration of federal Head Start, state-level school readiness, voluntary prekindergarten, and special education programming. With the exception of preschoolers receiving special education services apart from the regular preschool classroom, all preschoolers in participating preschool programs were invited to participate in the study. Table 1 provides a summary of programs and participants.

### 2.2. Design

As random assignment was not possible, a quasi-experimental design (non-equivalent pretest–posttest) was used in this study. There were two independent variables. The first was preschool approach. Participating preschool programs were assigned to one of three groups (approaches) based on the use of nature-based practices. Group assignment was informed by two strategies. The first strategy entailed teachers completing the Preschool “Nature-ness” Categorization Rubric (see Table 2). This rubric has items that cover curriculum and instructional practices, nature-related teaching and learning practices, teacher roles, and indoor and outdoor environments. Thus, while not every item is unique to nature-based programs or specifically describes a nature-based practice, collectively the items are intended to provide general descriptions of the three approaches and allow for assigning programs into the three categories. This rubric was used for a similar purpose in prior studies [37,45]; see [45] for additional information on the development and field testing of the rubric.

For each of the thirteen items, teachers selected which of the three responses best reflects their classroom/instructional practices or environment. Responses are scored on a three-point scale, with the column on the left receiving three points, the column in the middle receiving two points, and the column on the right receiving one point. The responses across the 13 items were scored and totaled, with higher total points reflecting greater incorporation of nature-based practices.

Regarding program assignments to the categories, the highest scoring cluster of programs was the Nature approach (indicating the most incorporation of nature-based practices relative to the other programs). The lowest cluster of programs was the Less Nature approach (characterized by less incorporation of nature-based practices relative to the other programs). The remaining programs with the middle clustering of scores were assigned to the Blended category (characterized by more incorporation of nature-based practices than the programs using the Less Nature approach). However, it is important to note that sorting programs into program approaches resulted not only from the numerical scoring. The second strategy used was asking the school district’s early education coordinator to review the rubrics and to ensure that the resulting categorization of programs reflected each program’s degree of incorporation of nature-based practices. The coordinator was frequently present in these classrooms and thus very familiar with the instructional approaches used in participating programs as well as their classroom environments. The coordinator’s review of the rubrics and categorization of programs was particularly important for programs that were numerically in between two categories. Further, this review was also important as teachers’ perceptions of the descriptions within the items may have varied from teacher to teacher and from the meaning intended to be conveyed through the rubric; there was also potential social desirability in their responses. Through this review process, the aim was to have programs clustered in a way so that the approach (category label) reflected the programs within that particular grouping and programs within a grouping were more similar to each other than to programs in the other categories. This process resulted in the program approach categories as shown in Table 1.

Programs in the Nature category emphasized environmental outcomes (such as respect for nature), as well as kindergarten preparation in the form of developing curiosity, positive attitudes toward learning, independence, and other social-emotional outcomes. These outcomes are fostered primarily through unstructured nature play and/or loosely guided, playful outdoor learning, with experiences guided by children’s interests that emerge during play. These playful, unstructured learning experiences occur in a variety of settings, from nature playscapes in an urban setting to more ‘wild’ natural areas. The school day is primarily child-directed. The teacher’s role in Nature programs includes setting the stage and modeling play, as well as facilitating play planning and reflection. Additionally, observing play is another role for teachers and is used toward understanding children’s interests as well as documenting learning.

Blended programs tended to have a greater emphasis on kindergarten readiness and early academics than Nature programs, with some integration of unstructured nature play. In Blended programs, there was relatively equal use of teacher-directed instruction and child-directed play, with both natural areas and school playgrounds used for outdoor play in Blended programs. While there is some emphasis on fostering children’s respect for nature in Blended programs, there is not the same degree of emphasis as in Nature programs.

Less Nature programs had a greater emphasis on conventional early academics and Kindergarten readiness. The majority of the day in Less Nature programs tended to be teacher-directed, and unstructured playtime tended to be indoors. Outdoor play was typically on the school playground, with the occasional use of a nature playscape or a less-maintained outdoor setting. In Less Nature programs, the teacher assumed a role that included primarily structuring, organizing, and leading activities for children.

In terms of the methodology, it is important to make clear that preschool programs were grouped relative to each other. While these rubric-based descriptions provide a sense of what the programs within each approach were like, it is important to be mindful that these categories represent a continuum as opposed to discrete categories and that the groupings in this study represented a limited range on this continuum.

The second independent variable was preschool type, with two levels: public and private preschools. The public preschool programs participating in this study were within a school district that integrates federal Head Start programming, state-funded school readiness, and voluntary prekindergarten. Of the study participants in public preschool programs, 85% were lower SES. There was no variation among the participants in the private school setting (all were higher SES). Consequently, SES was not a variable, as initially planned. Thus, the levels of the independent variable are stated as public and private for ease of writing, yet these levels more accurately represent a combination of SES and preschool type. Public preschool, in this study, is more accurately described as publicly funded preschool settings in which the majority of children served are of lower SES backgrounds; private preschool refers to preschool programs that are not publicly funded and serve students from higher SES backgrounds.

### 2.3. Construct and Measures

The dependent variables were the posttest levels of cool and hot executive control, as measured by the Preschool Self-Regulation Assessment (PSRA) [46], and posttest level of attention/impulse control, as measured by the PSRA Assessor Report (PSRA-AR) [46]. When assessing SR, it is recommended to specify which concept of SR a researcher’s construct and measure represents, differentiating among the following concept options: domain-general SR (all top-down and bottom-up aspects of SR); top-down SR (both simple and complex top-down processes); basic top-down processes (the lower level, basic, and early developing components of top-down SR in the context of an immediate stimulus); complex top-down processes (the high-level executive function processes oriented toward future conflict or challenge, rather than immediately cued conflict); and bottom-up processes (automatic, stimulus-driven, and non-cognitive) [1]. The executive control (EC) measures of SR used in the study at hand reflect the conceptualization of SR as basic top-down cognitive processes.

EC is similar to cognitive control [1] and relevant to the present demands made on young children in a preschool setting. Two types of EC are distinguishable theoretically and neurally [47]. Cool EC (CEC) involves an array of increasingly organized, flexible, goal-directed cognitive processes in response to non-affective (non-emotional) stimuli, whereas hot EC (HEC) is enacted in situations in which affective and motivational processes figure in more prominently, or in other words, in situations of emotional arousal [47]. Thus, CEC and HEC differ based on the predominant mechanism involved, cognitive for CEC and cognitive and affective/motivational for HEC [6]. Children draw upon CEC for classroom demands such as taking turns or staying focused in the presence of an interruption, and HEC is drawn upon in scenarios such as not taking a toy that belongs to another child, not becoming angry when a task becomes too challenging, and the ability to delay gratification [47]. While there appear to be some common processes across hot and cool tasks, as they both involve dorsal anterior cingulate cortex, anterior insula, and lateral and medial prefrontal cortex regions, research suggests tasks with emotional stimuli also draw on the amygdala, more rostral areas of the anterior cingulate cortex and medial prefrontal cortex, and orbitofrontal cortex, confirming separability of hot and cool EC, even with children [48].

The PSRA [46] in its original form consists of seven structured tasks to tap CEC and HEC in preschool-aged children, with tasks originating from [49,50]. The subsequent testing of the instrument led to a refined, shortened battery of four tasks, which provides a reliable and valid measure of hot and cool EC (see [47] for further information on the instrument’s reliability and validity). The two CEC tasks, Pencil Tap and Balance Beam, require children to filter competing stimuli, and the two HEC tasks, Toy Wrap and Toy Delay, tap effortful control to delay gratification (see Table 3).

In addition to the direct assessment that yielded performance data, the PSRA-AR was used, which provides a global picture of children’s emotions, attention, and behavior throughout the assessor–child interactions of the PSRA [46]. The PSRA-AR provides complementary but different information about a child’s SR than the information gained through direct assessment tasks of the PSRA [51]. The PSRA-AR has two dimensions, attention/impulse control and positive emotion. For the study at hand and its focus on SR, the 15 items measuring attention/impulse control were used, which has a reported reliability of 0.92 [52]. Items are scored on a four-point Likert scale ranging from 0 to 3. Item scores are summed for a total score, with higher scores reflecting higher levels of SR.

### 2.4. Data Collection Procedures and General Analytic Strategy

With site permission from the participating preschool programs and approval from the Institutional Review Board for the Social and Behavioral Sciences at the University of Minnesota, parent consent was obtained for participating children. Teachers completed a summary sheet regarding the children’s age in months, gender, and SES/eligibility for free or reduced tuition; race and ethnicity were not collected, due to the lack of variation and to avoid being able to identify specific participants. The four PSRA tasks were administered to children individually, with the PSRA-AR completed for each child immediately following the conclusion of the PSRA tasks. The instruments were administered twice, once at the beginning of the year (as pretests) and again at the end of the year (as posttests).

Raw scores were calculated for each task as directed in [46]; see Table 3. Per [52], raw scores were then converted to Z scores, which allowed the two CEC tasks to be averaged to yield a composite CEC score, and likewise for the two HEC tasks averaged for a composite HEC score. The PSRA-AR items were totaled into a summed score and then also converted to a Z score, so that all three dependent variables were in the same unit, that is, SD, with a mean of 0 and an SD of 1 [53].

General linear modeling (3 × 2 ANCOVA) was conducted to evaluate the main effect of preschool approach (Nature vs. Blended vs. Less Nature); the main effect of preschool type (public vs. private); and the interaction effect on HEC, CEC, and attention/impulse control. Pretest score, age, and gender served as covariates, and the Bonferroni adjustment procedures were used to control for Type I errors. Planned simple main effects analyses were conducted considering the study’s focus on effective and accessible SR interventions toward supporting SR, particularly among children of lower SES in public preschool settings.

## 3. Results

### 3.1. Cold Executive Control (CEC) Results

A two-way ANOVA was performed to evaluate the effects of preschool approach and preschool type on CEC, with covariates of pretest, age, and gender. The adjusted means and standard deviations are presented in Table 4. The results indicated no significant main effect for preschool approach, F(2, 106) = 1.45, *p* = 0.24, partial η^2^ = 0.03; no significant main effect for preschool type, F(1, 106) = 1.93, *p* = 0.17, partial η^2^ = 0.02; and no significant interaction between preschool approach and preschool type, F(2, 106) = 2.46, *p* = 0.09, partial η^2^ = 0.04. These results suggest the three preschool approaches, as well as the two preschool types, were of similar effectiveness in terms of CEC, when accounting for pretest level of CEC, age, and gender, and that the effectiveness of approach (Nature vs. Blended vs. Less Nature) did not vary by preschool type (private vs. public).

### 3.2. Hot Executive Control (HEC) Results

A two-way ANOVA was performed to evaluate the effects of preschool approach and preschool type on HEC, with covariates of pretest, age, and gender (see Table 4 for the adjusted means and standard deviations). The results indicated no significant main effect for preschool approach, F(2, 105) = 1.92, *p* = 0.15, partial η^2^ = 0.04; no significant main effect for preschool type, F(1, 105) = 0.01, *p* = 0.92, partial η^2^ < 0.01; and no significant interaction between preschool approach and preschool type, F(2, 105) = 0.42, *p* = 0.66, partial η^2^ = 0.01.

While the two-way interaction between preschool type and SES was not significant for HEC, the observed power of this interaction term was low (0.11). Consequently, it may likely have been too low to detect potential significance. Further, the plots of the estimated marginal means of HEC posttests visually suggested the presence of an interaction effect. As such, to further examine the potential interaction effect between preschool approach and type, follow-up analyses of simple main effects were conducted.

The first planned follow-up analysis focused on pairwise differences in posttest means among approaches within the public preschool setting, given the study’s aim of supporting SR, particularly among children of lower SES in public preschool settings, and in light of studies suggesting the potential for nature play to support SR. Pairwise comparisons indicated that when controlling for pretest, age, and gender, the public preschool children who experienced the Nature approach had significantly higher HEC posttest means than their peers in public preschool where the Less Nature approach was used, MD = 0.49, SE = 0.23, *p* = 0.02; see Table 4 for adjusted means and standard errors. This suggests the effectiveness of the Nature approach for public preschool programs serving primarily lower SES children.

While not the study’s primary focus, a second planned follow-up analysis was conducted to explore pairwise differences in posttest means among the three approaches within the private preschool setting. None of the pairwise comparisons were significant, suggesting the three approaches were similar in terms of their impact on HEC for higher SES children in private preschool settings (see Table 4 for adjusted posttest means and standard errors).

### 3.3. Attention/Impulse Control Results

A two-way ANOVA was performed to evaluate the effects of preschool approach and preschool type on attention/impulse control (from the assessor report of the PSRA), with covariates of pretest, age, and gender. The adjusted means and standard deviations are presented in Table 4. The results indicated no significant main effect for preschool approach, F(2, 106) = 2.18, *p* = 0.12, partial η^2^ = 0.04; no significant main effect for preschool type, F(1, 106) = 2.54, *p* = 0.11, partial η^2^ = 0.02; and no significant interaction between preschool approach and preschool type, F(2, 106) = 0.94, *p* = 0.39, partial η^2^ = 0.02.

The two-way interaction between preschool type and SES was not significant, yet the observed power of this interaction term was low (0.19) and the plots of the estimated marginal means of attention/impulse control posttests suggested an interaction effect. Consequently, follow-up analyses of simple main effects were conducted, which allowed for examining the potential interaction between preschool approach and type.

As was the case for HEC, the first follow-up analysis of simple main effects focused on pairwise differences in posttest means among approaches within the public preschool setting. Pairwise comparisons indicated that when controlling for pretest, age, and gender, the public preschool children who experienced the Nature approach had significantly higher attention/impulse control posttest means than their peers in public preschool where the Less Nature and Blended approaches were used, MD = 0.66, SE = 0.27, *p* = 0.02 and MD = 0.63, SE = 0.28, *p* = 0.03, respectively. This suggests the effectiveness of the Nature approach for public preschool programs serving primarily lower SES children. The second planned follow-up analysis explored pairwise differences within the private preschool setting and yielded no significant pairwise comparisons among approaches. This suggests Nature, Blended, and Less Nature approaches do not differ in terms of their effectiveness on attention/impulse control for higher SES children in private preschool settings (see Table 4 for adjusted posttest means and standard errors of preschool approach by preschool type).

## 4. Discussion

### 4.1. Discussion of Findings and Research Recommendations

The results suggest incorporating nature-based practices into preschool was effective for supporting HEC and attention/impulse control among lower SES children in public preschool. Children in public preschool where a Nature approach was used had significantly higher HEC posttest levels than preschoolers in programs that had more minimal use of nature-based practices (Less Nature approach). They also had significantly higher attention/impulse control posttest levels than preschoolers in programs using the Blended and Less Nature approaches. Given the importance of SR on short-term outcomes, such as school readiness, as well as its foundational role in promoting well-being across the lifespan, this finding is noteworthy. The further significance of this finding is supported by research linking delayed gratification (one component of HEC) in early childhood to academic achievement and social-emotional capabilities in adolescence, and even health outcomes and career achievement in adulthood [54]. Additionally, HEC is integral to affective decision-making, which is associated with children’s emotional development and higher-order thinking [55]; a lack of this ability is associated with behavioral problems and risky behavior as children proceed throughout life [56]. Thus, the potential for a nature-based approach to support preschoolers’ SR is of significance, particularly due to the cascading effects of low SR on children’s future development.

The results at hand are consistent with [39], which found kindergartners’ use of green schoolyards supported SR development, with more frequent visits (daily v. weekly) and visits of greater duration associated with greater gains in SR. In contrast, several prior studies on constructs relevant to SR suggest some incorporation of nature-based practices (a Blended approach) may be sufficient. For example, in recent studies on young children’s protective factors associated with resilience [45] and executive function [37], preschools using a Blended approach were more impactful than those using a Less Nature approach; the use of a Nature approach did not lead to even stronger outcomes than the Blended approach. The findings from this study similarly support the assertion in [45] that the needed “dose” of nature-based practices likely varies based on the outcome variable at hand. For some outcomes, the Blended approach may be sufficient, and for others, such as HEC and attention/impulse control, a greater “dose” of nature-based practices potentially may be needed for significant impact.

Regarding explanations for the effectiveness of the Nature approach on HEC and attention/impulse control, the research literature offers some possibilities. The characteristics of the Nature approach are unstructured play in nature or naturalized outdoor settings. Thus, sociodramatic play [31], play-oriented physical activity [33], and child autonomy [34], which are features of unstructured nature play, may account for this impact of nature-based practices on SR. Additionally, interventions to support SR and executive functioning are more effective if children’s needs across developmental domains are also addressed [27,57], and thus it is perhaps unsurprising that preschools using nature-based practices may be associated with higher levels of SR, as they tend to be more holistic in terms of developmental focus. Yet these characteristics and features are likely present to some extent within the Blended approach, and thus, they may not sufficiently explain why the Nature approach was significantly more effective than the Less Nature approach, while the Blended approach was not.

One distinguishing feature of Nature programs relative to Blended programs is the greater duration of unstructured nature playtime. In their study of Kindergarteners’ use of green schoolyards, in which greater frequency and duration were associated with greater gains in SR [39], researchers raise the possibility that routine exposure to nature may not just provide immediate, temporary attention restoration, but actually build the base for SR. Referencing prior work on the “instorative” benefits of exposure to nature [58], which is the state of being more balanced, less impacted by stressful events, they posit that nature may deepen children’s base capabilities for regulating their emotions and behaviors [39]. They align their findings with the “more is better” recommendation from research [59] that suggests positive outcomes are proportional to the degree range of greenness or nature-based instruction. While the study at hand lacks the design and precision from which to infer a needed “dose”, the findings here, alongside [39], suggest that SR may be one of the outcomes where “more” of the nature-based practices is necessary for significant SR impact.

Another potential difference between the Nature approach and the Blended and Less Nature approaches is maturity and depth of play. Mature play, or play that is more advanced and fully developed, is associated with higher levels of SR [35]. Mature play is characterized by object substitutes that eventually become replaced using gestures and words to invoke imaginary objects, rich role play that goes beyond the portrayal of basic roles to more complex relationships among the roles and reciprocal actions, and high-quality play scenarios that integrate many themes and span the time of many days or even weeks [60]. Mature play is no longer common in early childhood classrooms; more common are stereotypical, less mature play scenarios, and a limited range of themes and roles [61]. Perhaps more troubling is research noting a regression in the level of play, with children reverting to less mature play as they progress through the preschool year [62]. Further, trends suggest a decline in play quality and quantity alongside a decline in young children’s SR [63].

Of the three approaches studied, the Nature approach seems most likely to afford this maturity and depth of play, due to longer periods of time that children engage in unstructured play coupled with the affordances of natural settings. This possibility is supported by a systematic review of qualitative research regarding nature play in early childhood education [64]; one theme that surfaces among the studies reviewed is the possibility of nature to not just afford play actions, but also play scripts, thereby sustaining the play story and leading to longer play episodes and greater child engagement. This review suggests nature-based environments support play that is of greater quality and maturity due to nature’s ability to “play back”, which is further described as a development-enhancing reciprocity and diversity afforded by nature-based environments when children have the opportunity to freely engage in unstructured, child-led, reciprocal interactions with nature, not just in nature [64]. They further allude to this reciprocal, playful engagement with nature that leads to high quality, deep play as requiring time—extended periods of unstructured time for children to tune into and engage with the affordances of natural settings [64].

Thus, it seems possible that the Nature approach supports more mature, high-quality play, with SR development beyond what unfolds in Blended and Less Nature classrooms, where play may not reach the depth afforded by the Nature approach. It is suggested that children may need to reach a certain level of play to benefit from it [63]. Perhaps this is true for HEC and attention/impulse control, whereas other relevant and desirable outcomes associated with play may have a lower threshold and be supported through some or less incorporation of Nature. Future research might explore maturity of play as a possible driver of the relationship between the Nature approach and SR, as well as the frequency, duration, and exposure to nature-based settings needed to yield this type of mature play.

Also needed is further research exploring how program characteristics may be interacting to support the development of SR. For example, in a study of over fifty publicly funded early childhood programs, researchers found benefits, as well as trade-offs, when more classroom time was primarily spent on teacher-directed, cognitively-focused activities [65]. They found that classrooms with a cognitive emphasis supported mastery of specific math and language skills, yet the time spent in directed instruction was associated with less social activity time and consequently less time for social interactions with peers. And while classroom activities such as block construction, dramatic and fantasy play areas, and sand and water play were not associated with increased peer social interactions nor increased social skills, the researchers speculated that limited time allotted for these activities did not allow children time to develop complex sociodramatic play sequences necessary for increased social skill development [65]. They further speculate that not only did the presence of adults directing children’s time reduce opportunities for sociodramatic play with peers, it even reduced the need for children’s use of independent strategies, thereby interfering with the progression in SR development from other regulation to self-regulation [65].

Greater child autonomy and peer social interactions are likely interwoven with the characteristics of long periods of unstructured playtime and mature play. Thus, beyond exploring the potential of the Nature approach to afford more mature play, future research might also investigate if child autonomy, peer social interactions, and maturity of play potentially influence (moderating or mediating) the relationship between preschool approach and SR. Similarly, further research might also explore the auditory stimuli afforded by nature settings to see if it influences or partially explains the relationship between the Nature approach and SR, in light of research linking exposure to nature sounds with reduction in mental stress and muscle tension [66], as well as research links among rhythmic movement, motor development, and SR [67]. Further research is also needed to explore why nature-based practices in the study at hand were more impactful on HEC (regulatory processing in contexts involving reward, emotion, and motivation) than CEC (contexts that are cognitive).

In terms of implications for practitioners and policymakers, two noteworthy aspects stem from these findings. First, while the preschoolers in the public Nature programs did not have significantly higher CEC posttest levels than their peers in the Blended and Less Nature public programs, nor did children in the private Nature preschool programs outperform Blended and Less Nature peers, they “kept up” in terms of regulatory abilities with their peers in classrooms where there was more of an emphasis on conventional early academics. Thus, the Nature approach supported what might conservatively be described as sufficient SR development, while providing a host of other meaningful cognitive, social-emotional, physical, and environmental outcomes evidenced in the literature [57] that support children as they proceed through school and into adulthood. In other words, considering SR’s predictability of school readiness and academic success, this study suggests children can spend a significant amount of their preschool day in unstructured nature play and still be cognitively and socially emotionally “ready for school”. This is consistent with [39] and a reference to the “null effects framework” advocated for within the environmental education literature, where findings of no difference in outcomes between more conventional academic programs and nature- or environment-based classrooms is interpreted as a positive, meaningful outcome.

Secondly, and perhaps of even greater significance, SR in should be supported in children of lower SES backgrounds and nature-based approaches should be made more accessible to a broader range of children, in light of the lack of economic and racial diversity among the current demographics of the Nature preschool movement [68]. Given the significant relationship between SR and SES [22], supporting SR development through early childhood education is particularly beneficial for children from lower SES families [23]. Thus, the findings from this study are encouraging, as they suggest that nature-based practices may be an effective means for supporting SR among lower SES children, thus providing not only another intervention option for consideration, but one that offers a host of other health, well-being, and developmental benefits across the domains as well. Further, these findings suggest nature-based practices can be successfully implemented within publicly funded programs, situated in urban settings, with participants of lower SES, and programs. This is particularly encouraging, as it suggests an avenue toward greater equity in terms of who experiences and ultimately benefits from nature-based practices.

### 4.2. Limitations

A quasi-experimental design was used for this study, which presents threats to the study’s internal validity. One of those is the possibility for preexisting differences in SR that may have influenced SR directly over the school year or interacted with other in- or out-of-preschool factors. Additionally, other between- and within-group differences, beyond the integration of nature-based practices, may have influenced SR, making it difficult to attribute SR outcomes to any one factor. Another threat to validity stems from the “nested” data, where participants were nested within classes and within preschool approaches. Multi-level modeling was not used, due to insufficient sample size at the program level and the groups were a fixed rather than a random factor [69], and consequently there could be inaccurate statistical estimates. A further limitation stems from the intervention being a continuum as opposed to discrete categories. While the group assignment process was guided by prior studies and utilized two strategies (the rubric and the review by the district’s early education coordinator), it was imperfect and an alternative process may have led to different results. However, despite these limitations stemming from the study design, this study does offer insight into the effectiveness of nature-based practices when delivered under usual conditions in a regular community setting, thus strengthening the study’s external validity [70].

## 5. Conclusions

Given its significant influence on school readiness and success, SR is a crucial skill in early childhood. It is also predictive of many outcomes across the lifespan, and thus of great interest to educators, policymakers, and researchers. This study assessed the effect of nature-based practices on preschoolers’ SR, conceptualized as cool and hot EC and attention/impulse control. Although more research is needed to determine a causal relationship, findings suggest that for public preschool, nature-based practices were more effective in supporting HEC and attention/impulse control than programs with less incorporation of nature-based practices. Thus, the integration of nature-based practices in early childhood education may be an effective way to support SR in lower SES children. This is relevant and significant, particularly given the relationship between SES and SR and the cascade of negative effects often initiated when children enter school with under-developed SR skills. Also, these findings are encouraging as they suggest the potential for greater equity in terms of who experiences and ultimately benefits from nature by extending the use of nature-based practices beyond the private into public preschool settings.

## Figures and Tables

**Table 1:** Summary of preschool and study participant data.

Preschool Approach ^a^	Program	Program’s Self-Reported Degree of “Nature-Ness” ^b^	Preschool Type	Study Participants’ Average Age (Mos.) ^c^	% of Female Participants	% of Lower SES ^d^ Participants	# of Study Participants
Less Nature	a	24	Public	53	31	93	13
	b	28	Public	54	67	100	9
	c	24	Public	56	71	93	14
	d	26	Private	54	58	0	12
	Subtotal						48
Blended	e	30	Public	54	67	47	15
	f	32	Public	52	42	92	12
	g	33	Private	52	43	0	14
	Subtotal						41
Nature	h	35	Public	54	42	100	12
	i	38	Private	51	50	0	14
	Subtotal						26
Total							115

^a^ The Less Nature category comprises preschools where there is less integration of nature-based approaches relative to the other programs; the Blended category has more integration of nature-based approaches than Less Nature; and the Nature category has the most integration of nature-based approaches relative to the other programs. ^b^ Scores on the Nature-ness Rubric have a possible range of 13–39; higher scores reflect higher levels of nature-based settings and practices. ^c^ Age in months at the time of the pretest. ^d^ Lower SES participants are income-eligible for free or reduced preschool tuition based on state and federal guidelines that are dependent on household size and income and based on U.S. poverty guidelines (see [43]). Participants reside in the upper Midwestern region of the United States; average median household income for this region is approximately USD 55,000, and community members are 89% White [44].

**Table 2:** Preschool “Nature-ness” Categorization Rubric.

Nature	Blended	Less Nature (Traditional)
Instructional focus is on both environmental outcomes (nature connection, respect for nature) and kindergarten preparation (developing curiosity, love of learning, problem-solving, independence, as well as other social-emotional outcomes).	Instructional focus is on both environmental outcomes (nature connection, respect for nature) and kindergarten preparation (early literacy and math, positive behaviors, and other social-emotional outcomes).	Instructional focus is on kindergarten preparation, including developing early literacy and math skills and fostering positive in-classroom behaviors, as well as other social-emotional outcomes.
Social and emotional learning (as well as other desired outcomes) is accomplished primarily through nature play and/or playful, guided outdoor learning, as well as teacher-guided negotiations.	Social and emotional learning (as well as other desired outcomes) is accomplished through a combination of developmentally appropriate direct instruction, curriculum materials, indoor play, outdoor play, and outdoor play in nature.	Social and emotional learning (as well as other desired outcomes) is accomplished through developmentally appropriate direct instruction and curriculum materials, as well as through play (primarily indoors).
More of the day is child-directed than teacher-directed.	Relatively equal use of teacher-directed activities and child-directed activity.	More of the day is teacher-directed than child-directed.
Classroom management toward positive behaviors emphasizes developing empathy and community.	Classroom management toward positive behaviors involves a combination of classroom expectations, classroom rules, and developing empathy and community.	Classroom management approach oriented toward classroom expectations and rules.
Substantial focus on child-directed nature play.	Some child-directed nature play is encouraged.	A small amount of child-directed nature play is encouraged.
Some teacher-guided nature learning outdoors (with a greater emphasis on child-directed playful learning when outdoors).	Some teacher-guided learning outdoors.	Small amount of teacher-guided learning outdoors.
Much impromptu nature learning based on what is found outdoors/in nature (including weather-related topics).	Some impromptu nature learning based on what is found outdoors/in nature (including weather-related topics).	Infrequent impromptu nature learning outdoors.
During outdoor playtime, the teacher primarily observes play toward understanding children’s interests and interactions, using this information to guide and enrich future outdoor learning and play.	During outdoor play, the teacher carries out a range of things, from actively guiding play or joining into play, to providing ideas for play, to observing children’s play in order to maintain safety and appropriate child behavior and interactions.	During outdoor play, the teacher primarily observes and/or actively guides play, maintaining safety and appropriate child behavior and interactions.
Time in the indoor classroom is primarily child-driven. The teacher sets up the indoor classroom with open-ended activities for children to choose from.	Inside, teachers lead small and/or large group activities along with providing time for child-directed play.	Inside, teachers structure, organize, and often lead activities for children. There is an emphasis on teacher-designed activities for children.
Emphasis on respect for nature and others (equal emphasis).	Emphasis on respect for nature and others, with slightly more emphasis on respect for others.	Emphasis on respect for others (and respect for nature as secondary).
Teachers allow children to work out conflicts on their own as much as possible.	Teachers balance child and teacher negotiation strategies to resolve conflicts.	Teachers provide guided negotiation when conflicts arise.
Indoor environment includes substantial nature content in wall displays, classroom materials, etc. Classrooms softly lit.	Indoor environment has some nature content. Classrooms brightly lit.	Indoor environment emphasizes other things relevant and of interest to preschoolers. Classrooms brightly lit.
Outdoor environment used is primarily an unmaintained, natural setting; a maintained natural play space is also available.	Variety of outdoor environments used, including unmaintained natural area, maintained naturalized outdoor play space, and outdoor playground.	Outdoor playground is primarily used when outdoors, with naturalized outdoor play space and/or a natural environment also available.

Note: Rubric from [37]; see [45] for more information on rubric development.

**Table 3:** PSRA tasks.

Task	Construct	Description	Scoring
Pencil Tap	CEC	Child is instructed to tap once when assessor taps twice, and twice when assessor taps one (16 trials).	Number of correct responses out of 16.
Balance Beam	CEC	Child is asked to walk on a balance beam. Then, child is asked to walk the balance beam again, this time slowly, and then a third time, as slowly as possible.	Difference between third trial and first trial, in seconds.
Toy Wrap	HEC	Assessor noisily wraps a surprise, asking the child not to peek for one minute.	Latency to first peek in seconds (up to 60 s).
Toy Delay ^a^	HEC	Child is asked to keep hands flat on the table and not retrieve toy hidden under a cup until timer beeps. Four trials, with progressively longer wait time, and a different toy hidden each trial.	Scored as level of waiting during timed wait time (scored from 1–5, with 5 indicating child waited for timer to beep with hands flat on table). Score is the average of four trials.

^a^ The original task was “Snack Delay” with candy hidden under cup, rather than toy. The decision to use a toy was to reduce risks relating to food allergies.

**Table 4:** Adjusted posttest means ^a^ and standard errors for CEC, HEC, and attention/impulse control by preschool approach and type.

Preschool Approach	Preschool Type	CEC Posttest Mean (SE)	HEC Posttest Mean (SE)	Attention/Impulse Control Posttest Mean (SE)
Less Nature	Public	−0.05 (0.10)	−0.16 (0.12)	−0.25 (0.14)
	Private	−0.17 (0.18)	−0.10 (0.21)	0.25 (0.25)
	Combined	−0.11 (0.10)	−0.13 (0.12)	−0.004 (0.14)
Blended	Public	−0.17 (0.11)	−0.04 (0.13)	−0.23 (0.16)
	Private	0.35 (0.17)	0.12 (0.19)	0.18 (0.23)
	Combined	0.09 (0.10)	−0.04 (0.12)	−0.02 (0.14)
Nature	Public	0.07 (0.17)	0.33 (0.20)	0.40 (0.23)
	Private	0.21 (0.16)	0.15 (0.19)	0.35 (0.22)
	Combined	0.14 (0.12)	0.24 (0.14)	0.38 (0.16)

^a^ Adjusted for pretest, age in months, and gender; standardized (Z) scores.

## Data Availability

The datasets presented in this article are not available because this data concerns young children and parents did not consent to inclusion of their children’s data in a dataset that may be shared with other researchers.
